# A new monitoring system for nutritional status assessment in children at home

**DOI:** 10.1038/s41598-023-30998-x

**Published:** 2023-03-13

**Authors:** Annamaria Zsakai, Dorina Annar, Beatrix Koronczai, Kinga Molnar, Petra Varro, Erika Toth, Szilvia Szarvas, Tamas Tauber, Zsolt Karkus, Dora Varnai, Agota Muzsnai

**Affiliations:** 1 Department of Biological Anthropology, Faculty of Science, ELTE, Eotvos Lorand University, Pazmany P. S. 1/C, 1117 Budapest, Hungary; 2 Health Promotion and Education Research Team, Hungarian Academy of Sciences, Budapest, Hungary; 3 Department of Department of Developmental and Clinical Child Psychology, Faculty of Education and Psychology, ELTE, Eotvos Lorand University, Budapest, Hungary; 4 Department of Anatomy, Cell and Developmental Biology, Faculty of Science, ELTE, Eotvos Lorand University, Budapest, Hungary; 5 Department of Physiology and Neurobiology, Faculty of Science, ELTE, Eotvos Lorand University, Budapest, Hungary; 6 Department of Microbiology, Faculty of Science, ELTE, Eotvos Lorand University, Budapest, Hungary; 7 Veres Palne Gymnasium, Budapest, Hungary; 8 Apaczai Csere Janos Gymnasium, ELTE, Eotvos Lorand University, Budapest, Hungary; 9 Department of Clinical Psychology and Addictology, Faculty of Education and Psychology, ELTE, Eotvos Lorand University, Budapest, Hungary; 10 North-Central Buda Center, New St. John’s Hospital and Clinic, Budapest, Hungary; 11 Pazmany P. s. 1/a, Doctoral School of Biology, Faculty of Science, ELTE, Eotvos Lorand University, Budapest, Hungary

**Keywords:** Epidemiology, Population screening

## Abstract

Regular monitoring of children’s nutritional status is essential to prevent micronutrient deficiencies, nutritional status abnormalities as stunting, wasting, overweight and obesity. Nutritional status assessment is usually performed by paediatricians by using anthropometry (body mass index, weight to height indices) and/or by body fat-mass measurement (bioimpedance analysis, dual-energy x-ray absorptiometry, computer tomography, etc.). Parents are also interested in but usually fail to evaluate their child’s nutritional status. To help the sufficient collaboration between the physician and parents a new nutritional status monitoring method is developed for families. The new monitoring system was developed under a paediatrician’s supervision by considering national and international recommendations, references as well as the anthropometric measurement possibilities at home. The model requires age, sex, body mass, height, waist circumference and hand circumference as predictor (input) variables of nutritional status, while (1) the centile values of the measured body dimensions, (2) body fat percentage and the centile of body fat percentage, (3) the nutritional status category (undernutrition, normal nutritional status, overfat/obese) can be predicted (outcome variables) by the new method. The predictive accuracy of the model for nutritional status category was 94.88% in boys and 98.66% in girls. The new model was developed for nutritional status assessment in school-aged children and will be incorporated in the healthy lifestyle module of ‘Teenage Survival Guide’ educational package to be developed by the Health Promotion and Education Research Team, Hungarian Academy of Sciences, Hungary. The new monitoring system could help the families to identify the early signs of malnutrition in children. Nutritional status assessment in children at home is suggested twice a year, and in case of suspicious nutritional status abnormality it is recommended to visit the general practitioner.

## Introduction

The obesity epidemic has become one of the most important health issues in this lifestyle transition leading to noncommunicable disease burdens. Obesity appears to be an unavoidable consequence of modern living^1^. Our perspective on obesity and unhealthy lifestyle should be reframed: “Being healthy—avoiding obesity is a life choice” should be a main message of health behaviour change programmes in our societies^2^. Moreover, obesity is such a serious medical condition that affects nowadays not only adults but also children and adolescents.

Beside obesity, underweight status also makes children much more vulnerable to somatic and mental illnesses. This nutritional status abnormality is still under-recognized and undertreated in spite of its irreversible and long-lasting health implications.

The identification of early signs of these two types of abnormal nutritional status is crucial. Usually, nutritional status assessment is done once a year in school-aged children in the public health system. However, to screen once a year for nutritional status abnormalities is not enough, since nutritional status can alter very intensively in children and adolescents due to the natural growth and developmental processes, and significant changes of lifestyle factors or health status can also result in nutritional status alterations.

Obesity and underweight are usually defined as body mass index (BMI) being over or under the age-dependent cut-off (critical) value (WHO)^7^. BMI is an inexpensive and easy screening method of nutritional status abnormalities; however, BMI is not an adequate indicator of percent of excess body fat^8^, BMI has serious limitations in nutritional status assessment. Body fat percentage (BF%) estimation is also a possible tool for estimating nutritional status, however it does also have some limits: (1) the body fat percentage estimation requires special equipment (e.g. dual-energy x-ray absorptiometry or bioelectrical impedance analyser), or (2) very few equations exist to calculate body fat BF% based on anthropometric measurements (usually circumferences, height and weight)^9,10^. (3) Moreover, the critical cut-offs of BF% has been determined only for obesity and only for adults^11^.

The parental involvement in children’s nutritional status assessment could sufficiently help the family-based lifestyle intervention activities. Since parents usually fail to evaluate their child’s nutritional status, an easy-to-use and sufficiently accurate method of children’s nutritional status assessment could help their evaluation^3^.

Therefore, a new monitoring system is suggested and introduced in details in the present paper for the monitoring of overfat/obesity and undernutrition in children aged between 6 and 18 years (and adults aged 18+) by using only very few, easy-to-perform body measurements. The new method does not require either children to remove clothing or special equipment.

## Subjects and methods

### Research design

#### Sampling method

Subject recruitment was done by using multilevel multistage sampling so as to obtain subjects that represented the diverse types of settlements and the age and sex distribution of Hungarian children aged between 7 and 18 years. The cross-sectional study was carried out in 2014–2015 in the Hungarian counties and in the capital. The main aim of the study was to collect information on children’s bone development and to analyze the accuracy of an ultrasonic method for skeletal maturity estimation compared to the standard radiographic methods used in the clinical practice. Body composition analysis and nutritional status assessment of children were also carried out in the auxological study.

The recruitment of participants was done by gaining access to children from the school authorities and by the official social media surfaces of Eotvos Lorand University. Sample size calculation was done by considering the margin of error, the population size of healthy children aged between 7 and 18 years (data were published by the Hungarian Central Statistical Office^4^, https://www.ksh.hu/stadat_files/okt/hu/okt0008.html), requested population proportion.

#### Informed consent

The printed subject information sheet (that provided information to the potential participants about the objectives and procedures of the study and sources of information) was delivered to the families via the school authorities. Written informed consent was obtained from the parents of children, and assent was obtained from the children as well.

#### Ethical approval

All experimental protocols were reviewed and approved by the institutional committee of the Research Ethics Committee of the Hungarian Scientific Research Fund (approval reference number: K-47073).

#### Data collection

Data on the level of habitual physical activity, the level of health status were collected by questionnaires (validated for the Hungarian population) through personal interviews. Anthropometric examinations were done in the schools.

### Body structural analysis

Body structural data of children (aged 7–18 ys, n: 1745, Table 1) were collected in a cross-sectional study in Hungary in 2014–2015^5^. The anthropometric measurements of children were performed using standardized techniques and standard equipment^6^. Body composition (body fat mass was expressed in the percentage of body weight, %) was estimated by body impedance analysis (by an InBody 720 analyser).

**Table 1:** Case numbers by age-groups and sex.

Age-group (years)	Boys	Girls
7	56	45
8	118	98
9	92	78
10	95	77
11	90	89
12	52	71
13	78	89
14	51	69
15	70	71
16	76	81
17	48	60
18	37	54
Together	863	882

### Statistical analysis

By following the WHO (1995) recommendation, the age-dependent body fat percentage cut-off values were used to define obesity on the basis of body fatness and constructed by using the smoothed centiles passing through the values of 25% BF% in boys and 35% BF% in girls at the age of 18 (Table 2). The centile pattern of body fat percentage was estimated by Cole’s LMS method^12,13^. Not only the cut-off values of centiles crossing 25% BF% in boys and 35% BF% in girls at the age of 18 were determined for screening obesity, but also the cut-off values of the 90th centiles for the screening of overfat nutritional status. In this case there was not any international or national recommendation or former method on how to construct critical values of overfat by using body fat percentage either in adults or in children. The 90th centile of BF% was used as the indicator of overfat (Table 2), since the 90th centile is usually used in epidemiological surveys in the case anthropometric dimensions or indices as cut-off criteria for abnormalities.

**Table 2:** The new body fat percentage cut-off values (%) for overfat and obesity in children and adolescents aged 7–18 years (P90: 90th centile, P96.95: 96.95th centile, P96.40: 96.40th centile values).

Age (years)	Boys		Girls	
	Overfat (P90)	Obese (P96.95)	Overfat (P90)	Obese (P94.60)
7.0	21.3	27.5	24.2	27.4
7.5	22.4	29.1	25.3	28.7
8.0	23.4	30.6	26.4	30.0
8.5	24.5	32.1	27.5	31.1
9.0	25.5	33.6	28.4	32.2
9.5	26.2	34.7	29.1	32.9
10.0	26.8	35.5	29.5	33.4
10.5	27.0	35.9	29.7	33.5
11.0	26.9	35.9	29.8	33.5
11.5	26.7	35.5	29.9	33.5
12.0	26.2	35.0	30.1	33.5
12.5	25.7	34.2	30.3	33.7
13.0	24.9	33.2	30.7	34.0
13.5	24.1	32.0	31.2	34.3
14.0	23.1	30.6	31.7	34.7
14.5	22.1	29.3	32.1	35.0
15.0	21.2	28.0	32.4	35.1
15.5	20.2	27.0	32.6	35.2
16.0	20.0	26.2	32.8	35.3
16.5	19.6	25.6	32.9	35.3
17.0	19.5	25.3	33.0	35.3
17.5	19.5	25.1	33.0	35.1
18.0	19.6	25.0	33.0	35.0

Children were divided randomly into the estimation subgroup and the cross-validation subgroup in both sexes (~ 50–50% of children were assigned to the two subgroups). Children’s data in the estimation subgroup were used to generate the multinomial logistic models for estimating nutritional status categories based on body fat percentage with anthropometric measurements and indices as predictors. Data of the cross-validation subgroup were used to check the accuracy of the multinomial logistic regressions in both sexes. More than 95% of children’s estimated (by the logistic regression equations constructed in the estimation subgroup) and observed nutritional status categories matched in the cross-validation subgroup.

Using the same group of predictor variables included in the estimation model, logistic analysis was also performed in the cross-validation subgroup in both sexes. Since the equations of estimation and cross-validation subgroups did not differ significantly (khi^2^ test was performed to compare the predicted nutritional status categories estimated by using the equations of estimation and cross-validation subgroups), the two subgroups were combined in both sexes, and the final models were estimated by using these combined samples in both sexes.

All statistic calculations and analyses were performed using SPSS (version 24, IBM Corporation, 2016). Significance was set at p = 0.05 level in the analyses.

In the beginning, regression analysis was performed to identify the possible predictors of body fat percentage among the body and extremity circumferences and weight to height ratios. However, none of the regression models predicted responses for new observations with higher R^2^ than 0.67. Therefore, as a next phase of the analysis, multinomial logistic regression was chosen to identify the strongest predictors of nutritional status category based on body fat percentage (not the exact BF% value, only its categorization into normal, overfat/obese nutritional status categories). Pearson correlation analysis was performed to determine the strength of association between body fat percentage and the easy-to-measure body dimensions (circumference) and easy-to-calculate indices (Table 3). In this preselection step the circumferences of waist, upper arm, lower arm, hand and the weight/height^3^ index were chosen for the multinomial logistic regression as possible predictors of nutritional status category in both sexes.

**Table 3:** Pearson correlation coefficients (r) of trunk and extremity circumferences and weight to height indices (p < 0.05 in each correlation) with body fat percentage.

Anthropometric dimensions, indices	Boys	Girls
Chest circumference (cm)	0.279	0.679
Waist circumference (cm)	0.429	0.751
Hip circumference (cm)	0.337	0.705
Upper arm circumference (relaxed, cm)	0.378	0.748
Lower arm circumference (cm)	0.236	0.641
Wrist circumference (cm)	0.247	0.593
Calf circumference (cm)	0.310	0.662
Ankle circumference (cm)	0.295	0.575
Hand circumference (cm)	−0.313	0.420
Weight/height ratio (kg/m)	0.338	0.726
Weight/height^2^ ratio (kg/m^2^)	0.574	0.796
Weight/height^3^ ratio (kg/m^3^)	0.782	0.713

Body fat percentage (estimated by InBody 720 equipment) was divided into normal and overfat/obese nutritional status categories by considering the new, age-dependent cut-off values of BF% (introduced by the present paper).

## Results

The description statistics of the logistic models that best approximate the relationship of the predictors to the BF% categories are presented in Table 4. The final model of multinomial logistic regression (best measures of goodness of fit, R^2^, kappa coefficient) revealed waist/height^3^ index, waist circumference and hand circumference (which circumference represents skeleto-muscular development and fatness at the same time, this might explain the negative correlation between hand circumference and body fat percentage in boys) as strong predictors of nutritional status in children (Table 4). By using the logistic model in boys 94.9%, in girls 98.7% of children’s estimated nutritional status (by using the logistic model prediction) matched the observed nutritional status category (undernutrition, normal nutritional status or overfat/obese status) based on BF% cut-off criteria.

**Table 4:** Descriptive statistics of multinomial logistic model to identify strongest predictor of nutritional status in children aged between 7–18 years.

	B	SE	p
Boys			
Intercept	−22.705	2.723	< 0.001
Waist circumference (cm)	0.178	0.041	< 0.001
Hand circumference (cm)	−0.664	0.180	< 0.001
Weight/height^3^ ratio (kg/m^3^)	1.514	0.187	< 0.001
R^2^ = 0.751, k = 0.765 (goodness of fit: p < 0.001)			
Girls			
Intercept	−14.354	2.255	< 0.001
Waist circumference (cm)	0.206	0.038	< 0.001
Hand circumference (cm)	−0.869	0.179	< 0.001
Weight/height^3^ ratio (kg/m^3^)	0.96	0.136	< 0.001
R^2^ = 0.671, k = 0.677 (goodness of fit: p < 0.001)			

B: estimated regression coefficient, SE: standard error of B, p: significance level of the regression; goodness of fit test: R^2^: the proportion of variance in nutritional status explained by the predictors, k: kappa coefficient, p: significance level of goodness of fit test.

The new method of nutritional status assessment—introduced in this paper—was tested in another sample of children (studied between 2016 and 2020 in the somatometric laboratory of the Department of Biological Anthropology, Eotvos Lorand University, Hungary; n: 299 boys and girls together, aged between 6–18 years), in which sample we also estimated body fat percentage by InBody 720 equipment and collected data on the anthropometric dimensions. The consistency between the nutritional status categorisation by using (1) BF% categories (the new body fat percentage cut-off values for overfat status, Table 2) and (2) the new method was 94.0% in the girls and 93.1% in the boys. The discrepancies were caused both the not identified overfat/obese status and the overfat/obese-labelled normal nutritional status cases in both sexes.

The new method is a suggestion for families to estimate children’s nutritional status in a very easy way. The nutritional status estimation of school-aged children should be done twice yearly at home. If the nutritional status is undernutrition or overfat/obese, and/or the nutritional status category changes between two examinations, a consultation with the general practitioners or a paediatrician is strongly recommended.

The healthy lifestyle module of the ‘Teenage Survival Guide’ educational package will include the new method of children’s nutritional status estimation. The instruction for the families on how to measure the required body dimensions will also be presented in the module. Furthermore, (1) the major medical comorbidities associated with nutritional status abnormalities (undernutrition, overfat, obesity) in childhood; and (2) the possibilities to intervene via diet or activity-related behaviours to prevent or treat childhood nutritional status abnormalities will be discussed in detail in the module.

### How to use the new method—instructions for parents to measure their children to get the required measurements for nutritional status assessment

Height and circumference measurements should be performed by an inelastic tape, while a weight scale is needed in the case of weight measurement.

#### Waist circumference

Measure the waist circumference of the child in the horizontal plane at the narrowest region between the chest and hips in standing position.

#### Hand circumference

Measure the circumference around the child’s left hand at the fullest part, where the fingers meet the palm (outstretched and closed fingers; exclude thumb!).

#### Height

Measure the child when she/he is standing with her/his back against a wall (legs are straight, arms are at sides, she/he stands with head, shoulders, buttocks and heels touching the wall), position the child facing forward, gently place a book on her/his head, mark with a pencil where the lower side of the book meets the wall. Measure the distance from the floor to the mark on the wall.

#### Weight

Measure the weight of the child (wearing normal indoor clothes) taken to the nearest tenth of a kilogram (or the nearest half kilogram).

Having these measurements, the examiner only has to (1) use them as input variables in the attached excel file and the excel file estimates the nutritional status of the studied child, or (2) use these measurements in the following equations (Table 4):

- boys: −22.705 + 0.178 × WC − 0.664 × HC + 1.514 × WH^3^;
- girls: −14.354 + 0,206 × WC—0,869 × HC + 0.96 × WH^3^,

where WC: waist circumference (cm), HC: hand circumference (cm), WH^3^: weight/height^3^ ratio (kg/m^3^). If the result equals or is smaller than 0, the estimated nutritional status is normal, if it is bigger than 0, the nutritional status is overfat/obese.

## Discussion

A new monitoring system for nutritional status assessment in children was introduced in the present paper. The regression analysis revealed that weight, height, waist/height^3^ index, waist circumference and hand circumference were the strongest predictor of nutritional status of children. Children were divided into nutritional status categories by considering the traditional anthropometric method, e.g. the international cut-off values of BMI, as well as by the cut-off values of body fat percentage constructed in the presented analyses. The accuracy of the new method for nutritional status category assessment was very high, namely 94.88% in boys and 98.66% in girls. Since the predictive equations were constructed by using body dimensions and mass components of Hungarian (representing Europid geographical variation/race) children, the body fat% estimation by using the kit is recommended in Europid children. The validation of the new method for other groups of children with non-Europid geographical variations will be done in 2023.

By entering the measurement data in the calculator, it estimates (1) the centile values of the measured body dimensions (by using the national reference centile distributions of height, weight, waist and hand circumferences^14^, (2) body fat% and the centile of body fat%, (3) the nutritional status category (undernutrition, normal nutritional status, overfat/obese status).

Nutritional status calculators are very rare all over the world, based only BMI calculation and categorisation, and mostly constructed to assess nutritional status in adults^15–18^. BMI-based assessment of nutritional status is not precise enough to distinguish between overweight caused by fat, muscular or their common excess. The absence of an accurate nutritional status assessment tool that uses only easy-to-measure body dimensions, which method can be used for nutritional status assessment children, was solved by the new monitoring system.

The new monitoring system will be incorporated into the healthy lifestyle module of a new online educational package to be constructed for health behaviour education in Hungarian children, but will be also available for the families interested in estimating their children’s nutritional status. Comorbidities of nutritional status abnormalities in childhood, the basic criteria for healthy living, advice on how to promote positive changes in diet and physical activity behaviours in children, the reliable online sources in Hungary on lifestyle factors, the registry of medical centres and public health systems will be also presented in the healthy lifestyle module of ‘Teenage Survival Guide’ educational package.

The nutritional status of children usually assessed once a year in the public health system. The nutritional status risk screening and assessment should be done more frequently in children, since body structure, body composition, nutritional status alter very intensively in children and adolescents during the body developmental processes. Moreover, the lifestyle and health status factors may also impact their nutritional status in a shorter interval.

The Health Promotion and Education Research Team (Eotvos Lorand University and the Hungarian Academy of Sciences, Budapest, Hungary, egyk.elte.hu) aims to provide an online platform to access educational resources for supporting school-aged children’s health behaviour development in connection with healthy lifestyle beside the topics of sex education, substance use, hygiene education, online activity and environment awareness. The healthy lifestyle module will be open from December 2023. The new monitoring system for the nutritional status assessment in children at home—introduced in the present paper—will be also incorporated in the web-based platform. Definitions of nutritional status categories, the morbidity and mortality risk of nutritional status abnormalities and the new tool for nutritional status assessment will be available in the healthy lifestyle module, too.

As final conclusions we could state that the main objectives have been met, (i) a new method for nutritional status assessment in children at home has been constructed, (ii) the predictive accuracy of the model for nutritional status category is high enough to encourage the families to use it; (iii) the nutritional status calculator has been available for the families on the website of the Health Promotion and Education Research Team. However, the Research Team is just in the beginning of the planned project, the full ‘Teenage Survival Guide’ must be available in 2 years. Our mission is to serve a useful educational package to the families and schools in Hungary to increase the level of knowledge of children in health behaviour, social media use and environmental awareness.

### Declarations

The Authors declare that (1) all methods were carried out in accordance with relevant guidelines and regulations, (2) all experimental protocols were reviewed and approved by the institutional committee of the Research Ethics Committee of the Hungarian Scientific Research Fund (approval reference number: K-47073), (3) informed consent was obtained from the legal guardians of all subjects, the participation was voluntary and data were anonymised and analysed for scientific purposes only.

## Data Availability

A Zsakai, the corresponding author should be contacted if someone wants to request the data from the present study.
